# Coronary Artery Bypass Grafting in Patients with Reduced Ventricular Function: The Devil Is in The Details

**DOI:** 10.21470/1678-9741-2024-0052

**Published:** 2025-02-05

**Authors:** Mesut Engin, Mustafa Abanoz, Ufuk Aydın, Yusuf Ata, Senol Yavuz

**Affiliations:** 1 Department of Cardiovascular Surgery, University of Health Sciences, Bursa Yuksek Ihtisas Training and Research Hospital, Yıldırım, Bursa, Turkey. E-mail: mesut_kvc_cor@hotmail.com; 2 Department of Cardiovascular Surgery, University of Health Sciences, Mehmet Akif Inan Training and Research Hospital, Sanliurfa, Turkey; 3 Department of Cardiovascular Surgery, University of Health Sciences, Bursa Yuksek Ihtisas Training and Research Hospital, Yıldırım, Bursa, Turkey; 4 Department of Cardiovascular Surgery, University of Health Sciences, Bursa Yuksek Ihtisas Training and Research Hospital, Yıldırım, Bursa, Turkey; 5 Department of Cardiovascular Surgery, University of Health Sciences, Bursa Yuksek Ihtisas Training and Research Hospital, Yıldırım, Bursa, Turkey

Dear Editor,

We have read the article by Senetra et al.^[1]^ entitled “Del Nido *vs.* Cold Blood Cardioplegia for High-Risk Isolated Coronary Artery Bypass Grafting in Patients with Reduced Ventricular Function” with great interest. First of all, we congratulate the authors for their good contribution. However, we would like to discuss some issues about high-risk coronary artery bypass grafting (CABG) in patients with reduced ventricular function.

The title of the mentioned article includes the expression “High-Risk Isolated Coronary Artery Bypass Grafting”. The European System for Cardiac Operative Risk Evaluation II (EuroSCORE II) value is the current and widely used preoperative risk scoring system in CABG^[2]^. In the study, EuroSCORE II median values of the patient groups were deter mined as 1.9 (1.1-2.6) and 2.2 (1.5-3.1)^[1]^. Based on what findings was the high-risk assessment made in the study? Today, to be clear, we believe that it has become more difficult to perform CABG on coronary artery patients with normal left ventricular function due to the increasing number of percutaneous coronary interventions. Having normal thyroid functions is an important condition for CABG operations. Abnormal conditions may increase the risks of postoperative complications. A study has shown that subclinical hypothyroidism also affects postoperative results^[3]^. In the mentioned study cohort of patients undergoing elective CABG, three patients had hypothyroidism^[1]^. In elective surgeries, we prefer to operate patients as euthyroid in our clinic. We would like to receive the valuable comments of the authors on this subject.

In CABG performed with cardiopulmonary bypass, ultrafiltration may be required in some patient groups. It is frequently needed in pediatric patients and adult patients with end-stage renal failure. An effect of its use to reduce the need for blood transfusion in elective CABG has not been demonstrated^[4]^. In their study, the authors applied intraoperative hemofiltration in 9% of the patients^[1]^. To what do the authors attribute this high rate? It was stated in the study that there were 11 patients suitable for the definition of “kidney disease”. Did these patients have advanced renal failure?

Del-Nido cardioplegia solution (dNCS) is a cardioplegia that can provide myocardial protection for up to 90 minutes when prepared as a single dose^[5]^. In our clinic, we generally prefer it in operations with prolonged cross-clamping (CC) times^[6]^. Also, we use dNCS in CABG in patients with multiple vessels and in whom all anastomoses are performed under CC^[7]^. In the mentioned study’s patient groups, CC durations were found to be 48.5 (36.5-56.0) and 53.0 (41.0-57.0)^[1]^. For example, in the patient group where dNCS was used, how was their case management in the patient whose CC time was 36.5 minutes?

Finally, although not statistically significant, the authors found that the rate of postoperative atrial fibrillation (PoAF) in the dNCS patient group was more than two times lower than in the other patient group. In order to comment on the risk of PoAF, many factors such as perioperative medical treatment status and lung diseases must be taken into consideration^[7,8]^.
